# Screening versions of the European Portuguese MacArthur-Bates Communicative Development Inventories Short Forms: development and preliminary validation

**DOI:** 10.3389/fpsyg.2025.1534392

**Published:** 2025-03-11

**Authors:** Marisa G. Filipe, Cátia Severino, Marina Vigário, Sónia Frota

**Affiliations:** Center of Linguistics, School of Arts and Humanities, University of Lisbon, Lisbon, Portugal

**Keywords:** MacArthur-Bates Communicative Development Inventories Short Forms, European Portuguese, screening versions, vocabulary assessment, infants, toddlers, typical development, language impairments

## Abstract

This study aimed to develop and validate the screening versions of the European Portuguese MacArthur-Bates Communicative Development Inventories Short Forms (EP CDI-SFs), intended to guide referrals for comprehensive language assessments in infants and toddlers. The first cohort, aged 8–18 months, included 1,293 typically developing children (*M*_age_ = 12.23, *SD* = 3.12, 50.2% male), 170 children at-risk for language impairments (*M*_age_ = 11.76, *SD* = 2.81, 45.9% male), and 39 children with Down syndrome (*M*_*age*_ = 12.28, *SD* = 3.40, 56.4% male), assessed using the EP CDI-SF Level I. The second cohort, aged 16–30 months, included 1,155 typically developing children (*M*_age_ = 23.45, *SD* = 4.07, 51.2% male), 181 children at-risk for language impairments (*M*_age_ = 23.23, *SD* = 4.31, 47% male), and 46 children with Down syndrome (*M*_age_ = 23.09, *SD* = 3.93, 69.6% male), assessed with the EP CDI-SF Level II. Through factor analysis, the 20 most psychometrically robust items from each form were identified and used to develop the new screening versions (EP CDI-Scr). Strong correlations between the EP CDI-SFs and EP CDI-Scr results for typically developing children, along with excellent internal consistency, supported the validity and reliability of the new tools. Furthermore, the EP CDI-Scr versions demonstrated excellent sensitivity and moderate specificity. They effectively distinguished between typically developing children, those at-risk for language impairments, and those with Down syndrome, confirming strong discriminant validity. These findings establish the preliminary validity, reliability, and effectiveness of the EP CDI-Scr, supporting timely referrals for comprehensive language evaluations.

## 1 Introduction

Language difficulties in children can have long-lasting effects on academic performance, social-emotional development, and behavior (Conti-Ramsden et al., 2018). Therefore, early identification of language impairments is crucial to ensure timely intervention. However, comprehensive language assessments are often challenging due to limited resources and the time-consuming nature of these evaluations (Fenson et al., 2000). A potential solution to optimize resources and address large populations is screening tools (Eadie et al., 2022). Screenings can be used by different stakeholders, including pediatricians, educators, and parents, to identify children at risk for language impairments, enabling timely referrals for more comprehensive assessments when necessary. This study aimed to develop and validate two screening tools to support the referral process for comprehensive language assessments of European Portuguese-learning infants and toddlers.

Traditional language assessment methods, such as language sampling and structured testing, exhibit considerable limitations when applied to children under the age of 2.5 years. As highlighted by Fenson et al. (2000), laboratory or clinic-based assessments are vulnerable to situational and temperamental factors, such as children’s anxiety or illness, which can lead to inconsistent performance or make testing infeasible. Additionally, these methods demand substantial resources and may be difficult to implement in clinical or research settings. Given these challenges, there is a pressing need for more efficient and effective tools for early language assessment.

The MacArthur-Bates Communicative Development Inventories (CDI) offer a cost-effective and valuable approach to assessing early language skills (Fenson et al., 1993; Marchman and Dale, 2023). The CDI applies a parent-centered approach, gathering information from caregivers about their child’s language abilities in natural settings, reducing many logistical issues associated with lab or clinic-based assessments. Caregiver-reported questionnaires are considered reliable and valid tools for collecting early developmental data. Given their frequent and close interactions with their child across various contexts, caregivers provide an unparalleled perspective on their child’s developmental progress, particularly in identifying potential developmental delays or disabilities, as international guidelines emphasize (e.g., World Health Organization and United Nations Children’s Fund (UNICEF), 2012). Indeed, parental reports have advanced our understanding of early language development, offering valuable insights into children’s profiles (e.g., Skarakis-Doyle et al., 2009; Frank et al., 2021; Fenson et al., 1994).

The CDI assesses various aspects of language development, including vocabulary comprehension, vocabulary production, and developmental milestones such as communicative gestures and word combinations (Fenson et al., 2000). This focus on vocabulary is well justified, as research showed strong correlations between vocabulary size and other aspects of early language development, such as gestural communication and grammatical skills (Bates et al., 1994; Fenson et al., 2007; Frank et al., 2021; Law and Roy, 2008). In the CDI, vocabulary size is measured using a checklist format, allowing caregivers to quickly recognize the words their child understands and uses.

Although the CDI is an effective tool, the original CDI Long Forms require considerable time to complete (typically 20–40 min), which may pose a limitation for research, educational and clinical settings. To address this, the CDI-Short Forms (SFs) have been developed. While these shorter versions provide less detailed data, they exhibited strong correlations with the original instruments, supporting their validity in measuring language abilities (Fenson et al., 2000). However, even the CDI-SFs can pose challenges, particularly in clinical-oriented research and clinical practice, where an efficient screening tool would be more advantageous. Early identification of language impairments through screening measures can mitigate negative effects on learning and behavior, improving health, academic performance, and social development (Wallace et al., 2015). The early years of life are a critical period for shaping children’s developmental trajectories (Shonkoff and Phillips, 2000), and research indicates that early intervention addresses language and communication challenges more effectively (McKean and Reilly, 2023; Weismer, 2000).

A screening tool for assessing early language skills could facilitate large-scale studies of language development and meet the specific demands of educational and clinical settings. By leveraging the strengths of parental-reported measures and addressing the limitations of traditional forms of assessment, a vocabulary-based screening tool holds great potential for enhancing early identification of risk for language impairments and improving language outcomes.

### 1.1 Present study

Two screening forms (CDI-Scr) were developed using data from typically developing children collected using the European Portuguese (EP) CDI-SFs (Frota et al., 2016) with two cohorts: one for infants aged 8–18 months and another for toddlers aged 16–30 months. Factor analysis was used to identify the most psychometrically robust items from each form, resulting in screening versions that can be easily administered in various settings. Authorization to develop the screening versions was granted by the CDI Advisory Board (see Table 1 for a comparison of the different CDI versions). The primary goals of this study were to develop and validate the EP CDI-Scr versions as reliable and effective screening tools for the quick identification of children at risk for language delays or disorders in early childhood. To achieve this, we assessed the psychometric properties of the new tools, including their validity and reliability. Specifically, we assessed the correlations between the performance of typically developing participants on the original EP CDI-SFs and the EP CDI-Scr, and the internal consistency of the EP CDI-Scr. Next, we evaluated the agreement between the EP CDI-SFs and the EP CDI-Scr using a criterion based on the 10th percentile. Children in the typically developing sample who scored below this threshold were classified as having lower performance. Based on this classification, sensitivity was defined as the proportion of children accurately identified as having lower performance, while specificity was defined as the proportion of children accurately identified as having typical performance. Finally, we examined discriminant validity by comparing the EP CDI-Scr scores of typically developing children with those of children at risk for language impairments and children with Down syndrome.

**TABLE 1:** The MacArthur-Bates Communicative Development Inventories (CDIs): comparison of the original long forms, European Portuguese (EP) Short Forms (EP CDI-SFs), and EP screening versions (EP CDI-Scr).

Original CDI – long forms			EP CDI-SFs		EP CDI-Scr	
**Form**	**Word and gestures**	**Word and sentences**	**Level I (infant)**	**Level II (toddler)**	**Infant**	**Toddler**
Age range	8–18 months	16–30 months	8–18 months	16–30 months	8–18 months	16–30 months
Objectives	Assess receptive and expressive vocabulary, communicative gestures, and symbolic gestures	Assess expressive vocabulary and early phases of grammar	Assess receptive and expressive vocabulary	Assess expressive vocabulary, word complexity and word combination	Screens for risk by assessing receptive and expressive vocabulary	Screens for risk by assessing expressive vocabulary
Number of items	396 vocabulary items separated into semantic categories 63 gestures	680 vocabulary items 5 items assessing the use of words 4 items for using suffixes to designate plurals and other grammatical forms (Word Endings/Part 1) 25 items for word forms 14 items for plurals and 31 for verbs endings (Part 2) A question on whether the child combines words. If yes, parents should list three of the child’s longest sentences 37 sentence pairs of word combinations based on complexity. Parents should select the member of each pair that best represents what the child produces, choosing the simpler or more complex sentence as applicable	90-word checklist	99-word checklist One question about word combination	20-word checklist	20-word checklist
Completion time	20–40 min to complete and 10–15 min to score		±10 and 5 min to score		3 – 5 min to complete and 2 min to score	

The EP CDI-Scr versions were expected to demonstrate strong correlations with the original EP CDI-SFs, maintain high internal consistency, exhibit high sensitivity, and effectively distinguish between typically and atypically developing children. They were thus anticipated to be a practical, reliable, and valid tool for early screening, enabling the quick identification of children at-risk for language delays or disorders. By facilitating early detection, these screening versions were intended to support timely referral for a more comprehensive evaluation of language and communication abilities in children who might exhibit language impairments. This will ensure that children needing further assessment receive appropriate attention, ultimately enhancing language acquisition and developmental support outcomes.

## 2 Methods

### 2.1 Participants

This study included two cohorts of monolingual European Portuguese-learning infants and toddlers. The first cohort, aged 8–18 months, consisted of 1,293 typically developing infants (*M*_age_ = 12.23 months, *SD* = 3.12, 50.2% male), 170 children at-risk for impairments in language and communication (defined as children born prematurely, i.e., <37 gestational weeks; with low birth weight, i.e., <2,500 g; low APGAR score at birth, i.e., <7; or a known familial risk for neurodevelopmental disorders, such as language disorders and autism spectrum disorders) (*M*_age_ = 11.76 months, SD = 2.81, 45.9% male), and 39 children with Down syndrome (*M*_age_ = 12.28 months, SD = 3.40, 56.4% male). The second cohort, aged 16–30 months, included 1,155 typically developing toddlers (*M*_age_ = 23.45 months, SD = 4.07, 51.2% male), 181 at-risk children as defined in the first cohort (*M*_age_ = 23.23 months, SD = 4.31, 47% male), and 46 children with Down syndrome (*M*_age_ = 23.09 months, SD = 3.93, 69.6% male). The typically developing participants were reported to have no medical conditions, according to information provided by their nursery school teachers or caregivers.

An analysis of the employment/educational status of parents/guardians^1^ showed that most participating families of the first cohort were classified as highly qualified (76.2%) or moderately qualified (17.3%), with a smaller proportion categorized as low qualified (2.6%). Additionally, 3.9% of parents for this age group were unemployed. In the second cohort, the pattern remained similar, with the majority of parents classified as highly qualified (61.3%) or moderately qualified (30.1%) and a smaller percentage in the low qualified category (3.9%). The unemployment rate for this group was 4.7%. This distribution likely reflects the sampling procedures and is consistent with findings from other CDI-SF norming studies, which show a tendency toward higher educational levels among caregivers (Fenson et al., 2000; Frota et al., 2016; Jackson-Maldonado et al., 2013; Simonsen et al., 2014).

### 2.2 Procedure

The study was approved by the Ethics Committee for Research of the School of Arts and Humanities of the University of Lisbon as part of the projects H21 (1_CEI2018), PLOs (13_CEI2019), and P2LINK (19_CEI2021). Participants were recruited through community outreach efforts, which included advertisements in hospitals, pediatric offices, nurseries, and community centers, as well as through invitation letters sent by the Lisbon Baby Lab to caregivers. Informed consent was obtained from all parents or guardians before the study. The EP CDI-SFs were administered during experimental sessions at the Lisbon Baby Lab or online. At the Lisbon Baby Lab, parents or guardians completed the questionnaires with the assistance of research assistants who provided guidance and support as needed in a quiet environment that minimized distractions. For those who completed the questionnaires online, detailed instructions and support were provided via email and phone to ensure accurate understanding and completion. The administration of the questionnaires took approximately 10 min.

### 2.3 Measures

The EP CDI-SFs (Frota et al., 2016) mostly assessed early vocabulary development. Their development followed the guidelines for the original short forms outlined in Fenson et al. (2000) and was informed by databases of spontaneously produced child speech and child-directed speech based on longitudinal corpora. Additionally, prior research on the acquisition of EP, the language-specific patterns, and parental feedback were also considered (see Frota et al., 2016 for a detailed description of the methodology underlying this tool). Specifically, the infant form (Level I) was administered to children aged 8–18 months, and the toddler form (Level II) was used for children aged 16–30 months. The Infant Form contains 90 vocabulary items with two separate columns, one for comprehension and another for both comprehension and production. Caregivers respond to each item by selecting whether the child understands each vocabulary item or both understands and says it. The Toddler Form includes 99 vocabulary items, and caregivers indicate whether the child produces each item. The final item in this form assesses the child’s ability to combine words, offering three response options: “not yet”, “sometimes”, or “often.”

The concurrent validity of the EP CDI-SFs was supported by the correlations between its scores and children’s performance in spontaneous speech samples (Frota et al., 2016). Content validity was ensured by the selection of the items according to research studies specific to European Portuguese and prior validation of the instrument’s earlier versions. The EP CDI-SFs have demonstrated high reliability, with a reported internal consistency of .99 (Frota et al., 2016).

The initial EP CDI-SFs were reduced to a 20-item screening version after considering the data collected from typically developing children. To ensure that the new screening versions maintain the most robust items for assessing vocabulary skills, a factor analysis was performed, one of the most effective methods for simplifying complex variables (Kerlinger, 1979).

The twenty items were selected for the new screening measure based on their strong factor loadings, indicating they were the most representative items of early vocabulary development. An Exploratory Factor Analysis (EFA) was performed using unweighted Least Squares (ULS), after assessing the assumptions for factor analysis. The Kaiser-Meyer-Olkin (KMO) measures of sampling adequacy were excellent (0.99), demonstrating the suitability of the sample size (Kaiser, 1970; Kaiser and Rice, 1974). Additionally, Bartlett’s Test of Sphericity showed significant results (*p* < 0.0001), confirming that the correlations between items were sufficient for factor analysis. The final 20 items selected are shown in Table 2. The screening tools are provided in the Supplementary Material.

**TABLE 2:** Factor loadings of the selected 20 items in the EP CDI-screening infant and toddler versions.

EP CDI-screening infant				EP CDI-screening toddler			
**Original item number**	**Description (Portuguese)**	**Description (English)**	**Factor loading**	**Original item number**	**Description (Portuguese)**	**Description (English)**	**Factor loading**
11	*carro*	car	0.750	32	*perna*	leg	0.829
17	*bolo*	cake	0.746	36	*garfo*	fork	0.810
22	*chapéu*	hat	0.782	40	*toalha*	towel	0.820
24	*meia(s)*	sock(s)	0.770	41	*cadeira*	chair	0.832
26	*cabeça*	head	0.756	42	*cama*	bed	0.823
27	*cabelo*	hair	0.786	43	*escada(s)*	ladder(s)	0.817
28	*dentes*	teeth	0.760	45	*quarto*	room	0.841
29	*olho(s)*	eye(s)	0.774	49	*chuva*	rain	0.823
33	*copo*	glass	0.767	50	*sol*	sun	0.806
34	*escova*	brush	0.747	53	*amigo*	friend	0.813
35	*garfo*	fork	0.750	56	*banho*	bath	0.810
38	*cadeira*	chair	0.790	62	*brinca/brincar*	play	0.855
39	*cama*	bed	0.751	66	*corre/correr*	run	0.828
41	*mesa*	table	0.781	69	*gosta/gostar*	like	0.824
45	*casa*	house	0.768	73	*salta/saltar*	jump	0.825
47	*flor*	flower	0.746	77	*bonito*	pretty	0.810
54	*menina*	girl	0.751	80	*frio*	cold	0.824
56	*chichi*	pee	0.753	83	*pequeno*	small	0.815
61	*cai/cair*	fall	0.748	94	*em cima*	on top	0.802
65	*gosta/gostar*	like	0.752	95	*muito*	much	0.817

### 2.4 Data analysis

All statistical analyses were performed using SPSS (Version 27). Descriptive statistics were computed for demographic variables. Inferential statistics, including correlation analysis (Pearson), internal consistency assessment (Cronbach’s alpha), and independent-sample *t*-tests, were used to evaluate the psychometric properties of the CDI-Scr tools. Acceptable internal consistency value has been suggested to be 0.7 and above (Streiner and Norman, 2008). A significance level of *p* < 0.05 was applied for all statistical tests. Additionally, sensitivity and specificity analyses were conducted to assess the screening tools’ effectiveness in identifying children with lower performance on the EP CDI-SFs. A 10th percentile criterion was applied to both assessments for this purpose.

## 3 Results

In the EP CDI-Scr Infant version, typically developing children presented the highest mean score (*M* = 7.34, *SD* = 8.40), followed by children with Down syndrome (*M* = 4.54, *SD* = 5.87) and at-risk children (*M* = 3.76, *SD* = 6.07). In the EP CDI-Scr Toddler version, typically developing children showed the highest mean score (*M* = 9.62, *SD* = 8.27), followed by at-risk children (*M* = 5.56, *SD* = 7.43), while children with Down syndrome had the lowest mean score (*M* = 0.80, *SD* = 2.39). The maximum score is 40 for the CDI-Scr Infant version (2 × 20 items) and 20 for the CDI-Scr Toddler version. Table 3 provides additional descriptive statistical information for comparisons among age groups for typically developing children.

**TABLE 3:** Descriptive statistics for the EP CDI-screening infant and toddler versions.

EP CDI-screening infant						EP CDI-screening toddler					
**Age**	***N***	***M***	**Mdn**	**SD**	**Min – Max**	**Age**	***N***	***M***	**Mdn**	**SD**	**Min – Max**
8	185	1.34	0	3.30	0 – 20	16	13	1.77	0	3.72	0 – 11
9	177	2.49	0	5.24	0 – 27	17	31	1.29	0	3.45	0 – 14
10	88	2.32	0	4.59	0 – 26	18	112	1.63	0	3.65	0 – 20
11	121	3.53	1	5.18	0 – 21	19	109	2.53	0	4.36	0 – 20
12	135	5.27	3	6.03	0 – 26	20	89	4.17	1	6.20	0 – 20
13	120	7.47	6	6.49	0 – 25	21	76	5.63	3	6.55	0 – 20
14	134	9.73	9	7.20	0 – 34	22	60	7.68	6.5	6.56	0 – 20
15	105	11.89	11.5	7.72	0 – 33	23	63	11.03	11	7.73	0 – 20
16	68	15.03	18	7.91	0 – 33	24	162	10.73	12	7.70	0 – 20
17	74	17.22	18	7.57	0 – 36	25	79	12.34	16	7.42	0 – 20
18	86	19.95	21	7.49	0 – 35	26	66	14.03	17	6.47	0 – 20
						27	40	17.10	20	4.35	7 – 20
						28	61	16.72	19	5.54	0 – 20
						29	66	17.26	20	5.15	0 – 20
						30	128	16.79	19	5.25	0 – 20

Number of typically developing children (N), Mean number of words (M), Median (Mdn), Standard Deviation (SD), Minimum (Min) and Maximum (Max), by age.

All skewness and kurtosis values were below | ± 3| and | ± 10|, respectively, suggesting no severe deviations from the normal distribution (Kline, 2005), satisfying the assumptions required for subsequent analyses.

The validity of the EP CDI-Scr Infant and Toddler versions was assessed by examining their correlations with the full EP CDI-SFs. For the Infant version, the correlation between CDI-SF comprehension and CDI-Scr was *r* = 0.948 (*p* < 0.001), indicating a strong relationship between the two versions. The correlation between CDI-SF production and CDI-Scr was *r* = 0.79 (*p* < 0.001), reflecting a strong relationship. For the Toddler version, the correlation between the CDI-SF and the CDI-Scr was *r* = 0.975 (*p* < 0.001), showing a strong correlation between the two tools.

The internal consistency of the screening versions was measured to ensure that the reduction in items did not affect the reliability of the assessments. For the infant screening version, internal consistency was excellent, with a Cronbach’s alpha = 0.967. Similarly, the toddler screening version exhibited excellent reliability, with α = 0.977.

The CDI-Scr Infant version showed a sensitivity of 99.4%, demonstrating its effectiveness in accurately identifying children with low performance on the CDI-SF. Its specificity was 60.2%, indicating moderate effectiveness in correctly identifying children with typical performance. Similarly, the CDI-Scr Toddler version had a sensitivity of 99.8%, effectively identifying children with low CDI-SF scores, and a specificity of 63.0%, moderately identifying children with typical CDI-SF performance.

The discriminant validity of the screening versions was assessed by comparing the scores of typically developing children to those of children at high risk for language impairments, and children with Down syndrome. Independent-sample *t*-tests were conducted to evaluate these differences. For the Infant version, scores were significantly different between typically developing children and at-risk children, *t*(263.06) = 6378, *p* < 0.001, with a small to medium effect size (Cohen’s *d* = 0.44). The difference was also significant for typically developing children and children with Down syndrome *t*(42.87) = 2.90, *p* = 0.006, with a small to medium effect size (Cohen’s *d* = 0.34). Similarly, for the Toddler version, significant differences were found between typically developing children and at-risk children, *t*(253.38) = 6.73, *p* < 0.001, with a moderate effect size (Cohen’s *d* = 0.50). The difference was also significant between typically developing children and children with Down syndrome *t*(97.25) = 20.59, *p* < 0.001, with a large effect size (Cohen’s *d* = 1.09).

## 4 Discussion

This study developed and validated the CDI-Scr versions for European Portuguese-learning infants and toddlers. These tools are designed to support the early identification of children potentially in need of comprehensive language evaluations. Item selection for the new screening tools was informed by factor analysis using data from typically developing cohorts aged 8–30 months, assessed using the EP CDI-SFs. As expected, findings showed strong correlations between the EP CDI-SFs and the EP CDI-Scr versions, as well as excellent EP CDI-Scr internal consistency, highlighting the validity and reliability of the new tools. However, in the younger cohort, correlations were stronger for vocabulary comprehension than for vocabulary production. This may be due to the high proportion of younger infants in the sample, as language comprehension typically develops earlier than production. Furthermore, the EP CDI-Scr versions effectively differentiated typically developing children from children at risk of language impairment or children with Down syndrome, demonstrating strong discriminant validity.

Our findings also indicated that the EP CDI-Scr versions exhibited excellent sensitivity, suggesting that the tools effectively identify children performing below the 10th percentile on the EP CDI-SFs. Nevertheless, the screening versions showed moderate specificity in accurately identifying children with typical performance on the EP CDI-SFs. This result contrasts with previous research, which often reports higher specificity than sensitivity in language screening instruments (Lavesson et al., 2018; Law et al., 2000). This observed discrepancy may arise from our methodology, which relied on caregivers’ vocabulary-based assessments. However, given the long-lasting effects of early language difficulties on later academic performance, social-emotional development, and behavior, (e.g., Conti-Ramsden et al., 2018) and the key importance of early identification to ensure timely intervention, we see the high sensitivity of the tools as an advantage, by limiting the number of false negative results and safeguarding that those children in need will ultimately get the necessary support.

This study has limitations that warrant further research. First, the CDI-Scr norming process is still ongoing, with efforts to equalize sample sizes across age groups and achieve a more balanced representation of parents from diverse socioeconomic backgrounds. Additionally, the analyzed age range restricts the EP CDI-Scr application to infants and toddlers within specified age groups, suggesting a need to test a broader age spectrum. The study’s cross-sectional design also limits insights into the tools’ predictive value over time. Longitudinal studies tracking language development could provide valuable data on the EP CDI-Scr’s effectiveness in predicting future language outcomes. Moreover, to further enhance the robustness and generalizability of the findings, it is essential to assess test-retest stability over time, and evaluate inter-rater reliability (for example, by comparing parent and preschool teacher ratings) to ensure consistency across different raters.

Furthermore, relying exclusively on caregiver reports may introduce variability due to differences in parental perceptions. Incorporating additional assessment methods or comparative analyses with other validated language evaluation tools could strengthen these findings. Future research should aim to cross-validate EP CDI-Scr data with comprehensive language evaluations.

Investigating the acceptability and feasibility of implementing the new screening tools among various stakeholders, including pediatricians, developmental psychologists, educators, and caregivers, is essential. Understanding how these stakeholders utilize the tools in their respective settings will provide insights into their practical application and potential barriers to implementation.

Although existing literature suggests a significant gap in empirical evidence supporting universal screening for language delays, particularly concerning the effectiveness of interventions for children screened for speech or language delays without prior concerns (Jullien, 2021), exploring the inclusion of the EP CDI-Scr as a universal screening tool may yield valuable insights. Research emphasizes the importance of regular, universal monitoring of language development during preschool, beginning in early infancy (McKean et al., 2016; Eadie et al., 2022). Given the well-documented impact of early intervention on developmental outcomes (Weismer, 2000), it is recommended that the EP CDI-Scr be implemented in routine pediatric or early educational check-ups to ensure children at risk for language delays are promptly identified and referred for further assessment. This approach could significantly reduce the prevalence and impact of language impairments, particularly in communities with limited access to specialized services.

Further research should also examine the EP CDI-Scr effectiveness across diverse demographic and linguistic groups. Such studies are essential to establish the tool’s applicability in varied contexts, ultimately promoting equitable access to language development screening for all children.

In conclusion, the EP CDI-Scr versions demonstrated robust validity and reliability in identifying infants and toddlers who may require comprehensive language evaluations. By developing and validating these screening tools, we have provided a feasible and practical approach for early identification, facilitating timely interventions to promote optimal language development. The tool’s quick and user-friendly design also promotes access to comprehensive language assessments, ultimately supporting improved developmental outcomes for children.

## Data Availability

The raw data supporting the conclusions of this article will be made available by the authors, without undue reservation.

## Footnotes

1 Professional categories followed the employment/educational status classification of the Portuguese Classification of Jobs (CNP, 2010, retrieved from http://cdp.portodigital.pt/profissoes/classificacao-nacional-das-profissoes-cnp).
