# Supplementary material for: Screening versions of the European Portuguese MacArthur-Bates Communicative Development Inventories Short Forms: development and preliminary validation
Source: Front Psychol. 2025 Mar 11;16:1534392. doi: 10.3389/fpsyg.2025.1534392 (PMC11956081; doi:10.3389/fpsyg.2025.1534392)
Supplement: Supplementary file 1 [file Data_Sheet_1.pdf]

Supplementary Material

CDI para o Português Europeu – Versão 'Screening' da Forma Curta: Nível I

Nome da criança: ____________________________ Sexo: ○ F ○ M

Data de nascimento: __/__/____ Data de hoje: ________________________

Instruções:
Para palavras que a criança compreende mas ainda não diz, assinale a primeira coluna (Compreende). Para palavras que a criança compreende mas também diz, assinale a segunda coluna (Compreende e diz). Se a criança usa uma forma diferente de dizer a palavra, assinale-a na mesma (ex.: 'gota' para gostar). No caso de palavras que podem ter formas femininas, masculinas, singulares e plurais, bem como diminutivos (ex.: 'menina', 'menino', 'flor', 'flores', 'florzinha') responda considerando qualquer uma das formas. Considere também as várias formas do mesmo verbo (ex.: 'cair', 'cai', 'caiu').

**Palavra**	Compreende	Compreende e diz
carro	○	○
bolo	○	○
chapéu	○	○
meia(s)	○	○
cabeça	○	○
cabelo	○	○
dentes	○	○
olho(s)	○	○
copo	○	○
escova	○	○
garfo	○	○
cadeira	○	○
cama	○	○
mesa	○	○
casa	○	○
flor	○	○
menina	○	○
chichi	○	○
cai/cair	○	○
gosta/gostar	○	○

CDI para o Português Europeu – Versão 'Screening' da Forma Curta: Nível II

Nome da criança: ___________________________ Sexo: ○ F ○ M

Data de nascimento: __/__/____ Data de hoje: ______________________

**Instruções:**
As crianças compreendem mais palavras do que dizem. Neste questionário, estamos interessados em saber as palavras que o/a seu/sua filho/a DIZ. Por favor, assinale as palavras que ouviu a criança dizer. Se ela diz a palavra de uma maneira diferente, assinale-a na mesma (ex.: 'gota' para gostar). No caso de palavras que podem ter diferentes formas, como masculino e feminino, singular e plural, ou diminutivos, responda considerando qualquer uma das formas (ex.: 'pequeno', 'pequena', 'pequeninos', 'pequeninhas'). Considere também as várias formas do verbo (ex.: 'correr', 'corre', 'correu').

**Palavra**	Diz
perna	○
garfo	○
toalha	○
cadeira	○
cama	○
escada(s)	○
quarto	○
chuva	○
sol	○
amigo	○
banho	○
brinca/brincar	○
corre/correr	○
gosta/gostar	○
salta/saltar	○
bonito	○
frio	○
pequeno	○
em cima	○
muito	○
